# Role of cysteine-rich angiogenic inducer 61 in fibroblast-like synovial cell proliferation and invasion in rheumatoid arthritis

**DOI:** 10.3892/mmr.2014.2770

**Published:** 2014-10-27

**Authors:** LI-GANG JIE, RUN-YUE HUANG, WEI-FENG SUN, SONG WEI, YONG-LIANG CHU, QING-CHUN HUANG, HONG-YAN DU

**Affiliations:** 1 Department of Chinese Medicine, Guangzhou General Hospital of Guangzhou Command of PLA, Guangzhou, Guangdong 510010, P.R. China; 2 HuaBo Bio-Pharmaceutic Institute of Guangzhou, Guangzhou, Guangdong 510010, P.R. China; 3 Department of Rheumatology, The Second Affiliated Hospital, Guangzhou University of Chinese Medicine, Guangzhou, Guangdong 510006, P.R. China; 4 School of Biotechnology, Southern Medical University, Guangzhou, Guangdong 510515, P.R. China

**Keywords:** rheumatoid arthritis, fibroblast-like synovial cell, cysteine-rich angiogenic inducer 61, cell proliferation, cell invasion

## Abstract

Cysteine-rich angiogenic inducer 61 (Cyr61) is a novel molecule that has been shown to be increased in the synovial tissues of patients with rheumatoid arthritis (RA). The present study was conducted in order to investigate the role of Cyr61 in the pathogenesis of RA. A human genome-wide gene assay was used to screen gene expression in synovial tissues obtained from four patients with RA and three patients with osteoarthritis (OA). To examine the role of Cyr61 in the phenotype of RA-fibroblast-like synovial (FLS) cells, Cyr61 expression in RA-FLS cells was knocked down using small interfering RNA (siRNA). Normal FLS cells transduced with lentiviral vectors encoding Cyr61 cDNA were used to further explore the effects of this molecule on FLS cell apoptosis, proliferation and invasion. The study found that the Cyr61 gene was highly expressed in the synovial cells from patients with RA compared with those from patients with OA. Downregulation of Cyr61 by siRNA led to impaired cell proliferation and invasion. Furthermore, it decreased the levels of matrix metalloproteinase (MMP)-3 and MMP-13, and induced apoptosis in RA-FLS cells. Conversely, overexpression of Cyr61 in normal FLS cells led to opposite effects. In conclusion, these results indicate that Cyr61 is capable of promoting RA-FLS cell proliferation and invasion via the suppression of apoptosis and the regulation of MMP expression. Therefore, Cyr61 may be a good target molecule for the treatment and prevention of RA.

## Introduction

Rheumatoid arthritis (RA) is a systemic autoimmune disease that affects multiple tissues and organs, although synovial joints are the main site of involvement (1). The basic pathology of RA has been identified as a disorder of inflammation in the rheumatoid synovium, in which the predominant cell type is the fibroblast-like synovial (FLS) cell (2,3). RA-FLS cells in the synovium are aggressively proliferative and invasive, and are known to attack cartilage, resulting in joint damage. Thus, the phenotype of RA-FLS cells is similar in a number of ways to that of tumor cells (3).

Cysteine-rich angiogenic inducer 61 (Cyr61) is a member of the CCN (also termed CTGF, Cyr61/cef10 and nov) gene family in which the expression can be induced by growth factors, cytokines, steroid hormones and certain drugs (4–6). Recently, Cyr61 has been found to be associated with a number of diseases related to chronic inflammation, such as RA, atherosclerosis, diabetes-related nephropathy and retinopathy, and several types of cancer (7). The induction of Cyr61 expression in RA is well-documented. For example, it has been shown that sirtuin-1/FoxO3a signaling is crucial for the induction of Cyr61 expression in RA synovial fibroblasts (8), and also that p53 is involved in the post-transcriptional regulation of Cyr61 expression via microRNA-22 (miR-22) (9). With regard to the role of Cyr61 in RA, it has been demonstrated that Cyr61 promotes neutrophil infiltration via upregulation of interleukin (IL)-8 production in FLS cells (10). In addition, Cyr61 has been shown to promote T helper 17 cell (Th17) development in RA via upregulation of IL-6 production by FLS cells (11). Furthermore, Zhang *et al* (12) demonstrated that Cyr61 is critical in IL-17-mediated proliferation of RA-FLS cells and may contribute to hyperplasia of the synovial lining cells and eventual joint destruction in patients with RA. Therefore, Cyr61 appears to be a crucial component of a vicious cycle formed by the interaction between infiltrating neutrophils, proliferating FLS cells and activated Th17 cells, in the development of RA (10).

Despite these recent advances, little is known about the role of Cyr61 in the phenotype of RA-FLS cells. The present study therefore explored the role of Cyr61 in FLS cell activities, including cell proliferation, apoptosis and cell invasion.

## Materials and methods

### Synovial specimens and genechip microarray

Synovial tissue specimens were obtained from four patients with RA and three patients with osteoarthritis (OA) who underwent arthroscopic surgery of the knee joint. All samples were collected from Guangzhou General Hospital of Guangzhou Military Command (Guangzhou, China). The tissue samples were stored in a liquid nitrogen tank prior to the experiments. Total RNA was extracted from all collected samples in order to synthesize cDNA. Subsequently, cRNA was amplified and labeled with biotin. A human genome-wide analysis was performed using the human genome U133 Plus 2.0 in a Genechip microarray hybridization oven 640 (Affymetrix Inc., Santa Clara, CA, USA), as described previously (13). All study protocols and consent forms were approved by the Institutional Medical Ethics Review Board of Guangzhou General Hospital of Guangzhou Military Command.

### Cell lines and cell culture

Normal FLS, RA-FLS and HEK293T cell lines were ordered from Cell Applications Inc. (San Diego, CA, USA). All cells were cultured in Dulbecco’s modified Eagle’s medium supplemented with 10% fetal bovine serum (FBS) at 37°C in a humidified atmosphere containing 5% CO_2_.

### Transient transfection of small interfering RNA (siRNA)

FLS cells were seeded at a uniform density into a 6-well culture plate and then incubated overnight to allow cells to attach to the plate. Cyr61-siRNA and non-target siRNA (control siRNA) were ordered from GenePharma Company (Shanghai, China). siRNA was transfected into the cells with the aid of Lipofectamine™ RNAiMAX reagents, according to the manufacturer’s instructions (Invitrogen, Carlsbad, CA, USA). Following 4 h transfection, the cells were recovered using fresh culture medium.

### Construction and production of lentivirus

The full-length human Cyr61 gene was cloned into the pCDH-CMV-MCS-EF1-copGFP lentiviral vector (System Biosciences, Mountain View, CA, USA) at the *Eco*RI and *Bam*HI sites using the following polymerase chain reaction (PCR) primers: 5′-TAGAGCTAGCGAATTCGCCACCATGAGCTCCCGCATCGC-3′, for CYR61-EcoRI-F and 5′-TCGCGGCCGCGGATCCTTAGTCCCTAA ATTTGGAATGTC-3′, for CYR61BamHI-R. Lentiviruses were produced in HEK293T cells using the pCDH-CMV-MCS-EF1-copGFP lentiviral vector encased in viral capsid encoded by three packaging plasmids (System Biosciences). The supernatant containing the viruses was collected at 48 h post-transfection and the viruses were concentrated as described previously (14). Normal FLS cells were infected with the lentiviruses at a multiplicity of infection (MOI) of five, in the presence of 8 μg/ml polybrene (Sigma-Aldrich, St. Louis, MO, USA).

### MTS assays and apoptosis analysis

FLS cell proliferation was determined by CellTiter 96^®^ AQueous One Solution Cell Proliferation assay (MTS assay; Promega Corporation, Madison, WI, USA), which was performed as previously described (15). Apoptotic cells were measured by a DeadEnd™ Fluorometric terminal deoxynucleotidyl-transferase-mediated dUTP nick end labelling (TUNEL system; Promega Corporation). Briefly, cells were seeded onto glass cover slides previously coated with 1% gelatin in 24 wells plates and cultivated to reach 80% confluency. Following siRNA or lentiviral vector transfection, culture medium was removed and fixation was performed with 4% neutral formalin in phosphate-buffered saline (PBS) for 25 min at 4°C. After washing twice with PBS, the cells were maintained in 70% ethanol at −20°C overnight. Cells were subsequently saturated and permeabilized with 0.2% Triton X-100 in PBS for 5 min. Subsequently, 100 μl buffer including 45 μl equilibration buffer, 5 μl nucleotide mix and 1 μl recombinant terminal deoxynucleotidyl transferase enzyme was added for 10 min. After washing for 15 min with saline sodium citrate buffer and twice with PBS, cells were incubated for DAPI staining at room temperature for 15 min in darkness. Acquisition of the images was performed with a fluorescence microscope (DMI6000B; Leica Geosystems, St. Gallen, Switzerland). Cells on each slide were counted in at least five fields, and the apoptosis ratio was taken as the number of positive cells divided by the number of DAPI-stained cells.

### Transwell in vitro invasion assays

Cell invasion experiments were performed using BioCoat Matrigel Invasion Chambers with 8 μm pores (BD Biosciences, Bedford, MA, USA), according to the manufacturer’s instructions. Briefly, cells were seeded at 2.5×10^4^ cells per well in serum-free medium overnight, and subsequently added to the upper chamber of a 24-well transwell plate. The lower chamber contained fresh culture media with 20% FBS as a chemoattractant. Cells were allowed to invade for 24 h at 37°C in the 5% CO_2_ atmosphere, and the chambers were then washed with PBS. Cells that did not invade through the membrane were removed, while the invading cells on the lower surface of the membrane were fixed with cold methanol, stained with 0.2% crystal violet and examined. The number of invading cells in each chamber was counted in at least five fields under a light microscope (OLYMPUS CKX41; Olympus Corp., Tokyo, Japan).

### Enzyme-linked immunosorbent assay (ELISA)

FLS cells were seeded at a uniform density into 6-well culture plates and incubated overnight. Following siRNA or lentiviral vector transfection, the culture supernatant was collected, centrifuged (2,000 xg for 10 min) and analyzed for the secretion of Cyr61, matrix metalloproteinase (MMP)-1, MMP-3, MMP-10 and MMP-13 with ELISA kits, according to the manufacturer’s instructions (Shanghai Westang Bio-Tech Co., Ltd., Shanghai, China).

### Reverse transcription-quantitative polymerase chain reaction (RT-qPCR)

Total RNA was extracted using TRIzol reagent (Invitrogen, Grand Island, NY, USA) according to the manufacturer’s instructions. cDNA was synthesized from 1 μg total RNA using a high capacity cDNA reverse transcription kit (GoScript^TM^ Reverse Transcription System; Promega Corp.). Aliquots of cDNA were used as the template for qPCR reactions containing gene-specific primers and SYBR Green qPCR SuperMix (Invitrogen). The following primer sequences were used: Forward: 5′-GGAAATCGTGCGTGACATT-3′ and reverse: 5′-CAGGCAGCTCGTAGCTCTT-3′ for β-actin; and forward: 5′-CTGAAGCGGCTCCCTGTTTT-3′ and reverse: 5′-GCACCTCACAAATCCGGGTT-3′ for human Cyr61. RT-qPCR was performed using the CFX96 Touch Deep Well™ Real-Time PCR Detection system (Bio-Rad Laboratories, Inc., Berkeley, CA, USA) using the following steps: 10 min at 95°C, followed by 40 cycles for 15 sec at 95°C and 15 sec at 60°C. The expression of target genes in the treatment and control groups was normalized using the reference gene β-actin, and the fold change in the expression of each target gene was calculated using the 2^−ΔΔCT^ method.

### Western blot analysis

Cells were washed with ice-cold PBS, collected and homogenized with radioimmunoprecipitation assay lysis buffer containing 1X PBS, 1% Nonidet P-40, 0.5% sodium deoxycholate, 0.1% SDS, and 1 mM phenylmethylsulfonyl fluoride (Beyotime, Beijing, China). Total protein was extracted and measured by the Bio-Rad protein assay (Bio-Rad Laboratories, Hercules, CA, USA). Equal quantities of protein (20 μg) were boiled for 10 min, separated by SDS-PAGE, and transferred to polyvinylidene difluoride membranes (Millipore, Billerica, MA, USA). The following antibodies were used for the western blot analysis: Mouse monoclonal antibody against Cyr61 (1:600) obtained from Santa Cruz Biotechnology (Santa Cruz, CA, USA) and rabbit polyclonal antibody against GAPDH (1:1,000) obtained from Cell Signaling Technology (Beverly, MA, USA). The secondary antibodies, affinity purified goat anti-rabbit immunoglobulin (Ig)G (H&L) and horse anti-mouse IgG (H&L) antibodies, conjugated to horseradish peroxidase, were purchased from Cell Signaling Technology (Beverly, MA, USA). Specific proteins were detected by using an enhanced chemiluminescence detection system (Clarity Western ECL Substrate; Bio-Rad Laboratories, Inc., Berkeley, CA, USA).

### Statistical analysis

For the human genome-wide analysis of synovial tissues, raw data processing, normalization and data analysis were performed with GeneSpring 7.31 software (Agilent, Santa Clara, CA, USA). Welch’s t-test analysis was subsequently used to select genes in which the expression varied at least 2.0-fold between RA and OA, with P<0.05.

For the other experiments, Student’s t-test was used to analyze the difference between two groups, whilst one-way analysis of variance followed by Dunnett’s test was employed for comparisons between three or more groups. Data are presented as the mean ± standard deviation for all statistical tests. P<0.05 was considered to indicate a statistically significant difference. Statistical analyses were performed using SPSS 16.0 statistical software (SPSS Inc. Chicago, IL, USA).

## Results

### Cyr61 is highly expressed in RA synovial tissues

**Figure 1:**
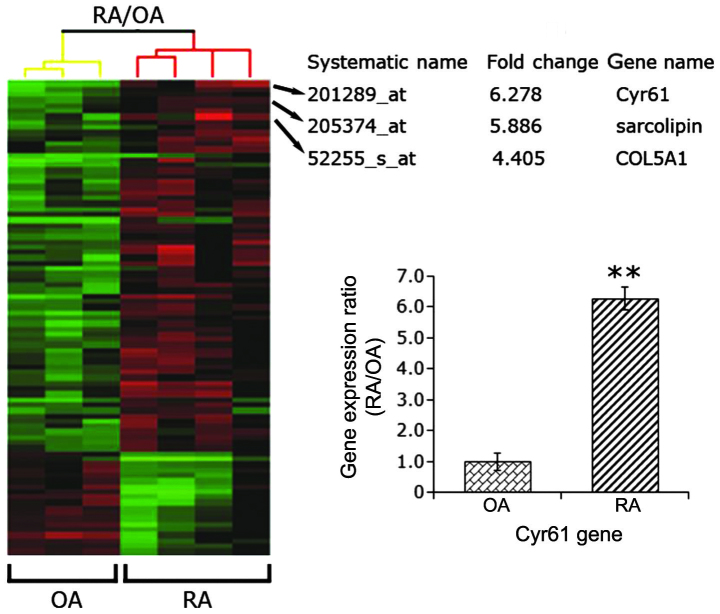
Human genome-wide analysis of RA and OA synovial tissues. Differential expression of at least two fold (P<0.05) between RA and OA samples was found in 338 genes, one of which was Cyr61. ^**^P<0.01, compared with OA synovial tissue. RA, rheumatoid arthritis; OA, osteoarthritis.

A previous study (12) using immunohistochemistry, RT-qPCR and western blot analysis found that Cyr61 is overexpressed in synovial tissue and FLS cells from RA patients compared with samples from disease-free control subjects. To confirm this result, four synovial specimens from patients with RA and three samples from patients with OA were collected. Using a human genome-wide analysis, the present study found that the gene expression of Cyr61 in samples from patients with RA was 6.28-fold that of samples from patients with OA (P<0.01; Fig. 1). In accordance with this result, Zhang *et al* (12) found that the level of Cyr61 was higher in synovial fluid samples from RA patients than those from normal controls. These findings suggest that Cyr61 may be involved in the pathogenesis of RA.

### Cyr61 promotes FLS cell proliferation

**Figure 2:**
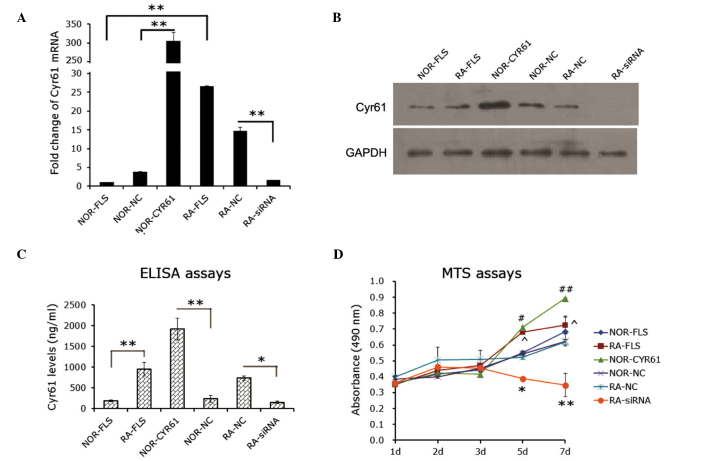
Role of Cyr61 in FLS cell proliferation. (A) Reverse transcription-quantitative polymerase chain reaction evaluation for the efficacy of Cyr61 siRNA or cDNA transfection in FLS cells. The mRNA level of Cyr61 in normal FLS cells was used as a control. ^**^P<0.01. (B) Western blotting evaluation for the efficacy of Cyr61 siRNA or cDNA transfection in FLS cells. Cyr61 (40 kDa) was detected using a mouse IgG1 monoclonal antibody specific for Cyr61. GAPDH served as the loading control. (C) Detection of levels of Cyr61 secreted into the culture medium using enzyme-linked imunosorbent assays. ^*^P<0.05 and ^**^P<0.01. (D) MTS assay detection of cell proliferation. Data are presented as the mean ± standard deviation of three independent experiments conducted in triplicate. ^*^P<0.05 and ^**^P<0.01, compared with RA-NC; ^#^P<0.05 and ^##^P<0.01, compared with NOR-NC; ^^^P<0.05, compared with NOR-FLA. RA, rheumatoid arthritis; FLS, fibroblast-like synoviocytes; siRNA, small interfering RNA; NOR-FLS, normal FLS cells; NOR-CYR61, normal FLS cells transduced with lentivirus vector encoding Cyr61 cDNA; NOR-NC, normal FLS cells transduced with control lentivirus vector; RA-NC, RA-FLS cells transfected with control siRNA; RA-siRNA, RA-FLS cells transfected with Cyr61-siRNA.

Due to the fact that Cyr61 was found to be overexpressed in synovial tissues, its involvement in the pathophysiological events associated with RA was investigated. RA-FLS cells were transfected with Cyr61-siRNA or control-siRNA, and effects on *in vitro* proliferation, apoptosis and invasion were determined. *In vitro* experiments were performed in RA-FLS cells without treatment and normal FLS cells. In addition, normal FLS cells were transduced with lentivirus vectors encoding Cyr61 cDNA or control lentivirus vectors. siRNA-mediated downregulation of Cyr61 resulted in a >80% reduction in Cyr61 mRNA expression in RA-FLS cells compared with normal FLS cells (P<0.01). Conversely, transduction of lentivirus vectors encoding Cyr61 led to a 304.43±24.14 fold increase in Cyr61 mRNA expression in normal FLS cells compared with normal FLS cells transfected with a control lentivirus (P<0.0001; Fig. 2A). The transfection efficacies were confirmed by western blot analysis (Fig. 2B). To further examine whether Cyr61 activity was affected by manipulation of its expression, the levels of Cyr61 secreted into culture media were assessed by ELISA assays. As hypothesized, the culture medium collected from RA-FLS cells contained higher levels of Cyr61 than normal FLS cells (P<0.0001; Fig. 2C). Levels of Cyr61 secretion in culture media were consistent with the status of Cyr61 expression; the level of secreted Cyr61 was elevated in normal FLS cells transfected with Cyr61 cDNA, whilst it was significantly decreased in RA-FLS cells transfected with Cyr61-siRNA (P<0.0001 and P<0.05, respectively; Fig. 2C).

Cell proliferation was determined at the time points after transfection indicated in Fig. 2D. The proliferation of RA-FLS cells was significantly greater than that of normal FLS cells. The MTS assays showed that the proliferation of RA-FLS cells for three, five and seven days was 105.9±0.2, 123.8±1.5 and 106.2±5.6% that of the normal FLS cells, respectively (Fig. 2D). The proliferative rates of RA-FLS cells transfected with siRNA were 74.3±0.9 and 56.2±7.4% those of RA-FLS cells transfected with control siRNA at five and seven days respectively, suggesting that the RA-FLS cell proliferative ability was significantly impaired by the introduction of Cyr61-siRNA. However, normal FLS cells, overexpressing Cyr61 showed enhanced cell proliferation at five and seven days (130.3±0.6 and 143.3±0.3% that of FLS cells transduced with control lentivirus vector, respectively; Fig. 2D).

### Cyr61 suppresses apoptosis in FLS cells

**Figure 3:**
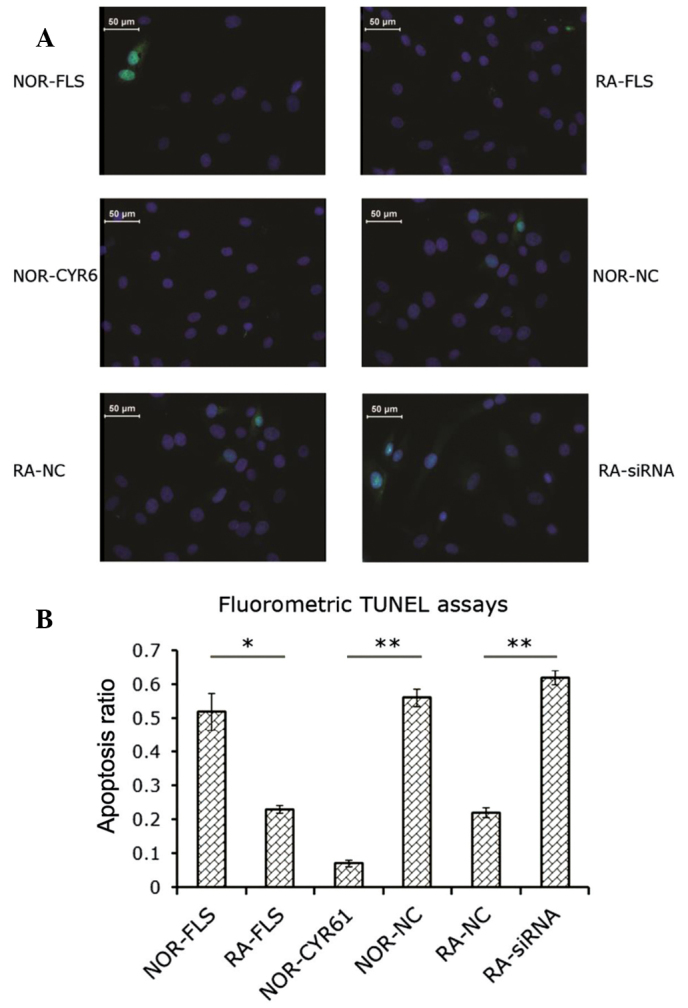
Role of Cyr61 in FLS cell apoptosis. (A) Fluorometric image of apoptotic cells. Blue, DAPI staining; Green, green fluorescent protein-positive staining for apoptosis. (B) Apoptosis ratio was calculated as indicated in the Materials and methods section. Data are expressed as the mean ± standard deviation of three independent experiments. ^*^P<0.05 and ^**^P<0.01. RA, rheumatoid arthritis; FLS, fibroblast-like synoviocytes; siRNA, small interfering RNA; NOR-FLS, normal FLS cells; NOR-CYR61, normal FLS cells transduced with lentivirus vector encoding Cyr61 cDNA; NOR-NC, normal FLS cells transduced with control lentivirus vector; RA-NC, RA-FLS cells transfected with control siRNA; RA-siRNA, RA-FLS cells transfected with Cyr61-siRNA; TUNEL assay, terminal deoxynucleotidyl-transferase-mediated dUTP nick end labelling.

The data obtained by the MTS assay implied that Cyr61 may have a pro-survival effect via promotion of FLS cell proliferation. Subsequent experiments were conducted to examine whether the status of Cyr61 expression in FLS cells affects apoptosis. Analysis of the proportion of apoptotic cells in each group was performed using the fluorometric TUNEL method. As shown in Fig. 3A, knockdown of Cyr61 in RA-FLS cells by siRNA led to increased cell apoptosis compared with RA-FLS cells without transfection (2.8-fold) or RA-FLS cells transfected with control siRNA (2.7-fold; P<0.0001 and P<0.01, respectively; Fig. 3B). The proportion of apoptotic RA-FLS cells was significantly less than that of normal FLS cells (P<0.05; Fig. 3B). Transduction of normal FLS cells with lentiviral vectors encoding Cyr61 cDNA decreased the fraction of apoptotic cells by 86.5±0.01% compared with normal FLS cells, and by 89.8±0.02% compared with normal FLS cells transduced with control lentiviral vectors (P<0.05 and P<0.01, respectively; Fig. 3B).

### Cyr61 facilitates FLS cell invasion

**Figure 4:**
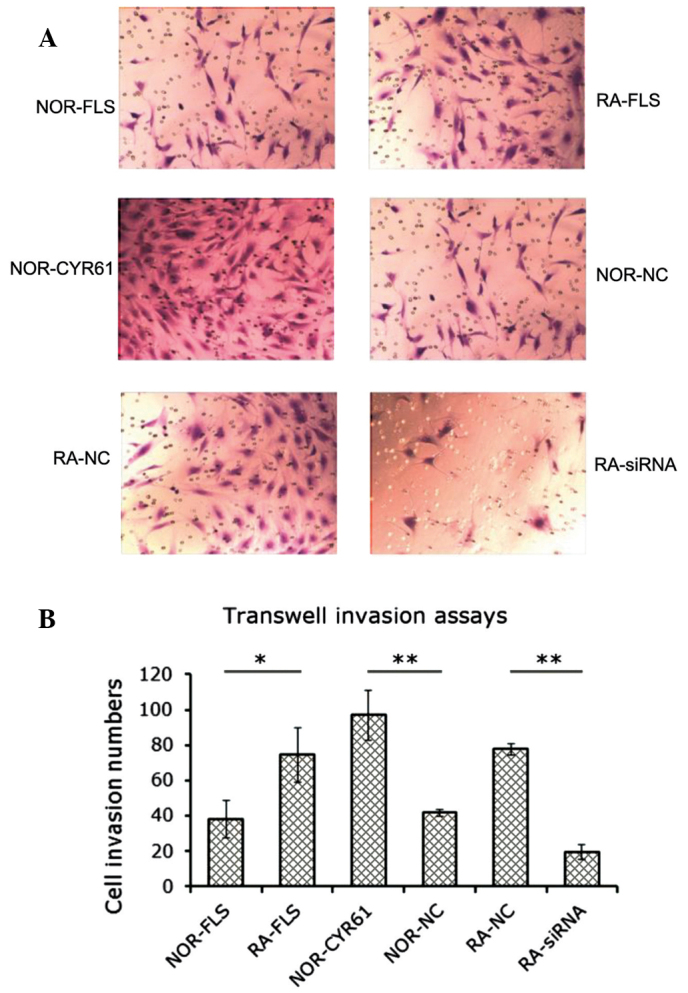
Role of Cyr61 in FLS cell invasion. (A) Image of the cells invading into the transwell. Original magnification, ×200. (B) The numbers of invading cells were calculated as indicated in Materials and methods. Data are expressed as the mean ± standard deviation of three independent experiments. ^*^P<0.05 and ^**^P<0.01. RA, rheumatoid arthritis; FLS, fibroblast-like synoviocytes; siRNA, small interfering RNA; NOR-FLS, normal FLS cells; NOR-CYR61, normal FLS cells transduced with lentivirus vector encoding Cyr61 cDNA; NOR-NC, normal FLS cells transduced with control lentivirus vector; RA-NC, RA-FLS cells transfected with control siRNA; RA-siRNA, RA-FLS cells transfected with Cyr61-siRNA.

The effect of Cyr61 on FLS cell invasion was investigated using transwell *in vitro* invasion assays. As shown in Fig. 4A, the number of RA-FLS cells or normal FLS cells overexpressing Cyr61 that were invasive was greater than that in the normal FLS cell group. In addition, as hypothesized, the number of the RA-FLS cells transfected with Cyr61-siRNA that were invasive was less that it was in the RA-FLS cells transfected with control siRNA. The number of invading cells was estimated as described, and is shown in Fig. 4B. RA-FLS cells showed markedly increased invasiveness compared with normal FLS cells, supporting the hypothesis that RA synovial tissue possesses a tumor-cell-like phenotype. Knockdown of Cyr61 in RA-FLS by siRNA resulted in a 73.1% reduction in the number of invasive cells (P<0.001), whereas overexpression of Cyr61 in normal FLS cells promoted cell invasion by 133.3% (P<0.001). These findings strongly suggest that Cyr61 leads to promotion of FLS cell invasion.

### Cyr61 regulates MMP-3 expression

**Figure 5:**
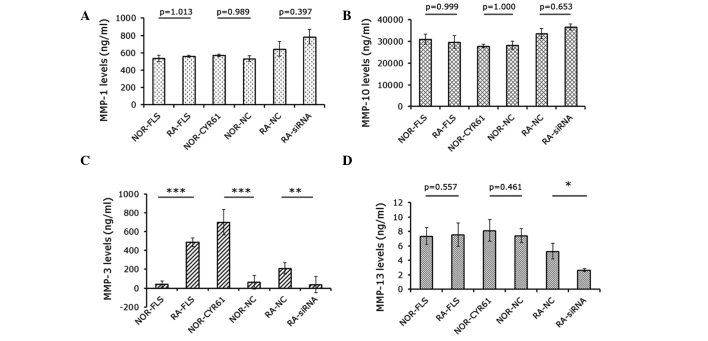
Effects of Cyr61 on expression of MMPs in FLS cells. Levels of (A) MMP-1, (B) MMP-10, (C) MMP-3 and (D) MMP-13. Data are presented as the mean ± standard deviation of three independent experiments done in triplicate. ^*^P<0.05, ^**^P<0.01 and ^***^P<0.001. MMP, matrix metalloproteinase; RA, rheumatoid arthritis; FLS, fibroblast-like synoviocytes; siRNA, small interfering RNA; NOR-FLS, normal FLS cells; NOR-CYR61, normal FLS cells transduced with lentivirus vector encoding Cyr61 cDNA; NOR-NC, normal FLS cells transduced with control lentivirus vector; RA-NC, RA-FLS cells transfected with control siRNA; RA-siRNA, RA-FLS cells transfected with Cyr61-siRNA.

MMPs are associated with cell proliferation, migration and invasion. Therefore this study sought to determine whether a change in Cyr61 expression affected the expression of MMPS, thereby potentially impacting on these processes. The levels of MMP-1, MMP-3, MMP-10 and MMP-13 secreted into the culture medium were detected by ELISA assays. As shown in Fig. 5A and B, the levels of MMP-1 and MMP-10 were not significantly altered by Cyr61. However, MMP-3 levels in RA-FLS cells were significantly higher than those in normal FLS cells (P<0.001). This difference was abrogated by downregulation of Cyr61 using siRNA (Fig. 5B). In addition, MMP-3 levels in normal FLS cells over-expressing Cyr61 were 15.6-fold higher than those in normal FLS cells and 10.9-fold higher than those in normal FLS cells transduced with control lentivirus vectors (P<0.0001; Fig. 5C). Furthermore, MMP-13 levels in RA-FLS transfected with Cyr61-siRNA cells were significantly reduced by 50.1% compared with cells transfected with control siRNA, and by 35.0% relative to RA-FLS cells without treatment (Fig. 5D).

## Discussion

The identification of unique and easily measurable biomarkers for use in RA diagnosis is a predominant aim of rheumatologists (16). Cyr61 was shown in the current study to be overexpressed in RA synovial tissue, synovial fluid and FLS cells (12) through the microarray and *in vitro* experiments. Synovial tissue, which is comprised predominantly of FLS cells, is the primary tissue targeted by the pathological processes involved in RA (3). This study focused on the role of Cyr61 in RA-FLS cells in terms of apoptosis, cell proliferation and cell invasion, in order to explore whether it may be used as a reliable marker in RA diagnosis.

The study used the dual approaches of ‘loss-of function’ and ‘gain-of function’. The data demonstrated that cell extracts and culture medium collected from RA-FLS cells contained higher levels of Cyr61 than normal FLS cells from individuals without RA. On the basis of these findings, Cyr61 expression was knocked down using siRNA in RA-FLS cells, and overexpressed in normal FLS cells by transducing lentivirus vectors encoding Cyr61 cDNA. A series of *in vitro* experiments were designed to examine the role of Cyr61 in FLS cell activity. It was found that RA-FLS cells had higher rates of proliferation and were more invasive than normal FLS cells, supporting the hypothesis that the phenotype of synovial cells is similar in a number of ways to that of tumor cells (3).

The current study showed that downregulation of Cyr61 in RA-FLS cells decreased cell proliferation, while overexpression of Cyr61 in normal FLS cells significantly increased cell proliferation. This suggests that Cyr61 is important in FLS cell proliferation. This result is in accordance with another study which showed that elevated levels of Cyr61 in RA synovial fluid promotes the proliferation of FLS cells and that this effect was abrogated by neutralizing Cyr61 (12). In addition, an analysis of apoptosis in the present study demonstrated that this process was induced by Cyr61-siRNA in RA-FLS cells, but was suppressed in normal FLS cells overexpressing Cyr61. This implies that Cyr61 promotes FLS cell proliferation in part by suppressing apoptosis.

Furthermore, the current study demonstrated that Cyr61 promoted cell invasion in FLS cells, as shown by the transwell invasion assays. The effects of Cyr61 on MMP expression were also examined. The MMP family are involved in a number of disease processes, including arthritis and tumor metastasis. They are also understood to be involved in certain cellular processes, such as cell proliferation, migration, differentiation, angiogenesis and apoptosis (17). MMP-3 is a key member of the MMP family. It is able to activate other MMPs, meaning it is crucial in the connective tissue remodeling process (18). During RA progression, FLS cells secrete MMPs, which degrade cartilage and bone. MMP-3 is the most important of these molecules (19). Measurement of active MMP-3 in clinical samples may thus provide information regarding the progression of rheumatoid diseases, and potentially also the response to treatment (20). Therefore, the data presented, showing that Cyr61 may significantly affect the level of MMP-3 secreted into the culture medium of FLS cells, suggests that Cyr61 promotes RA-FLS cell proliferation and invasion at least in part through regulation of MMP-3 expression. Notably, although MMP-13 levels were decreased by the introduction of Cyr61-siRNA into RA-FLS cells, they were not affected by overexpression of Cyr61 in normal FLS cells, suggesting that the effect of Cyr61 on MMP-13 expression may be dependent on cell context.

In conclusion, this study demonstrated increased levels of Cyr61 in synovial tissues and FLS cells from patients with RA. Cyr61 may act as a promoter for RA-FLS cell proliferation and invasion via suppression of apoptosis as well as the regulation of MMP-3 expression. Despite considerable efforts over a number of years, current therapeutic strategies for RA treatment remain unsatisfactory (21). Further *in vivo* studies are required to determine whether Cyr61 may be a candidate for therapeutic intervention.
