# Role of cysteine-rich angiogenic inducer 61 in fibroblast-like synovial cell proliferation and invasion in rheumatoid arthritis

**DOI:** 10.3892/mmr.2022.12901

**Published:** 2022-11-28

**Authors:** Li-Gang Jie, Run-Yue Huang, Wei-Feng Sun, Song Wei, Yong-Liang Chu, Qing-Chun Huang, Hong-Yan Du

Mol Med Rep 11: 917–923, 2015; DOI: 10.3892/mmr.2014.2770

Following the publication of the above article, an interested reader drew to the authors’ attention that Fig. 4A on p. 921, showing the results from cell migration assay experiments, featured a pair of duplicated data panels. After having consulted their original data, the authors have realized that Fig. 3A on the same page, showing the fluorometric images of apoptotic cells, also contained a pair of duplicated data panels. These errors in the presentation of these figures arose inadvertently as a consequence of selecting the wrong images for the ‘RA NC’ data panel in Fig. 3A and the NOR-FLS data panel in Fig. 5E.

The revised versions of Figs. 3 and 4 are shown on the next two pages. All the authors approve of the publication of this corrigendum, and the authors are grateful to the Editor of *Molecular Medicine Reports* for granting them the opportunity to publish this. The authors regret their oversight in allowing these errors to be included in the paper, and also apologize to the readership for any inconvenience caused.

## Figures and Tables

**Figure 3.**
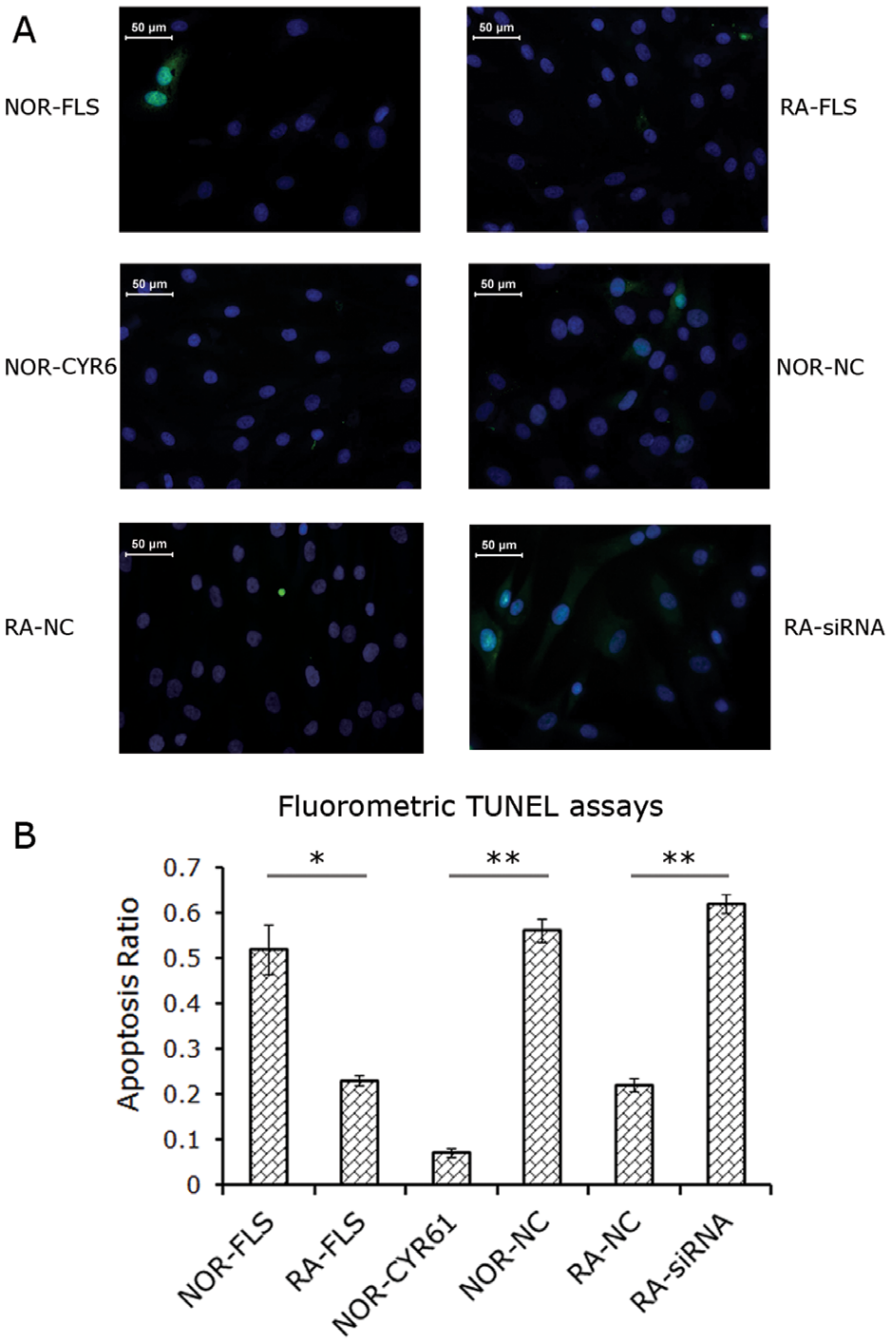
Role of Cyr61 in FLS cell apoptosis. (A) Fluorometric image of apoptotic cells. Blue, DAPI staining; Green, green fluorescent protein-positive staining for apoptosis. (B) Apoptosis ratio was calculated as indicated in the Materials and methods section. Data are expressed as the mean ± stan- dard deviation of three independent experiments. *P<0.05 and **P<0.01. RA, rheumatoid arthritis; FLS, fibroblast-like synoviocytes; siRNA, small inter- fering RNA; NOR-FLS, normal FLS cells; NOR-CYR61, normal FLS cells transduced with lentivirus vector encoding Cyr61 cDNA; NOR-NC, normal FLS cells transduced with control lentivirus vector; RA-NC, RA-FLS cells transfected with control siRNA; RA-siRNA, RA-FLS cells transfected with Cyr61-siRNA; TUNEL assay, terminal deoxynucleotidyl-transferase-medi- ated dUTP nick end labelling.

**Figure 4.**
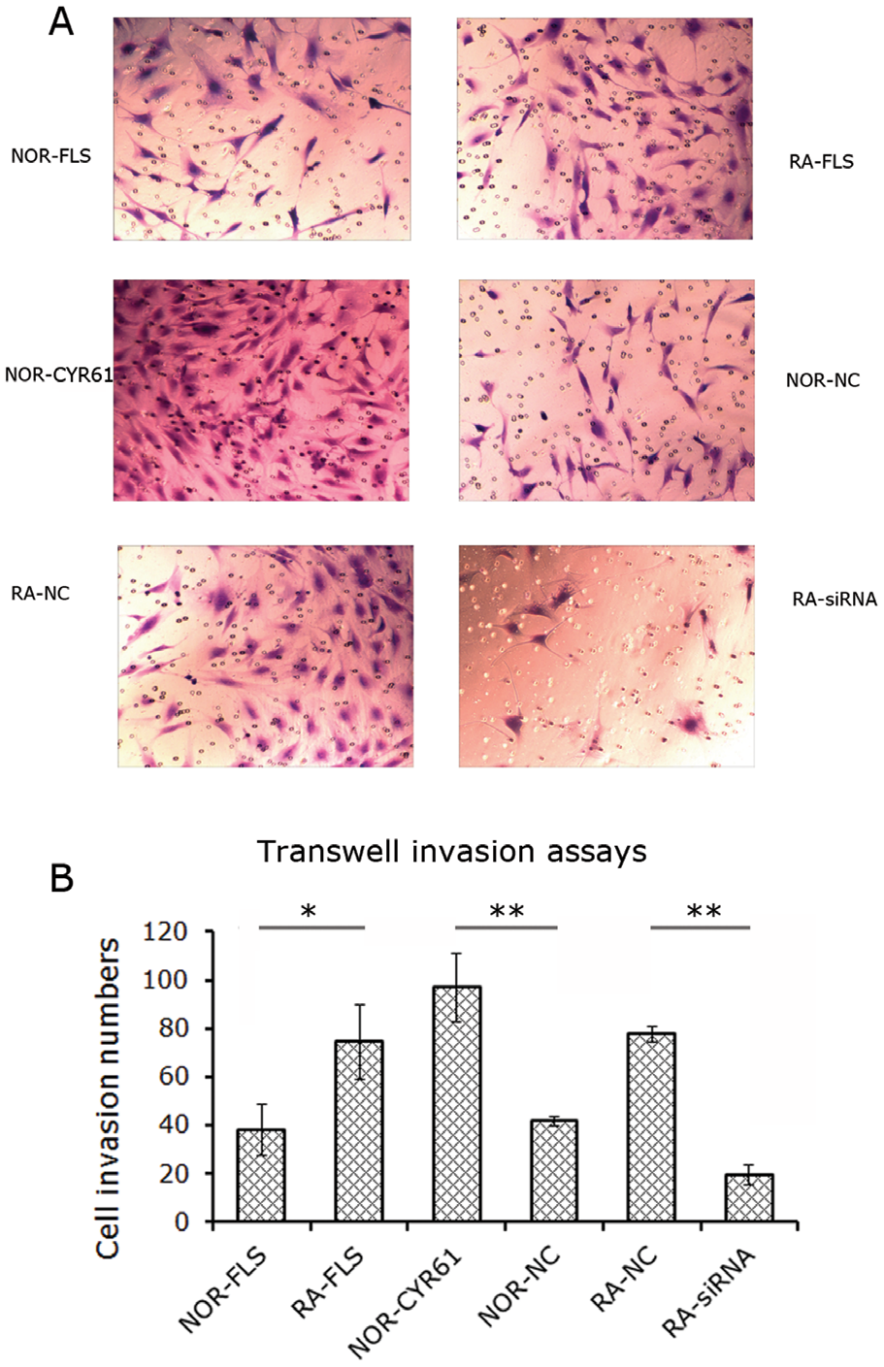
Role of Cyr61 in FLS cell invasion. (A) Image of the cells invading into the transwell. Original magnification, ×200. (B) The numbers of invading cells were calculated as indicated in Materials and methods. Data are expressed as the mean ± standard deviation of three independent experi- ments. *P<0.05 and **P<0.01. RA, rheumatoid arthritis; FLS, fibroblast-like synoviocytes; siRNA, small interfering RNA; NOR-FLS, normal FLS cells; NOR-CYR61, normal FLS cells transduced with lentivirus vector encoding Cyr61 cDNA; NOR-NC, normal FLS cells transduced with control lentivirus vector; RA-NC, RA-FLS cells transfected with control siRNA; RA-siRNA, RA-FLS cells transfected with Cyr61-siRNA.

